# Tobacco two-pore calcium channel 1a is localised at the tonoplast, but acts on events at the plasma membrane

**DOI:** 10.1007/s00709-025-02118-1

**Published:** 2025-10-02

**Authors:** Qiong Liu, Lena Seidler, Peter Nick

**Affiliations:** Molecular Cell Biology, Joseph Gottlieb Kölreuter Institute for Plant Sciences, Karlsruhe Institute of Technology, Fritz-Haber-Weg 4, 76131 Karlsruhe, Germany

**Keywords:** Actin, Auxin, Calcium, Hypersensitive response, Tobacco BY-2, Two-pore calcium channel

## Abstract

Calcium is a central signal regulating a plethora of cellular events. Specificity is brought about by spatio-temporal patterns, so-called signatures that are established by the activity of calcium channels residing in the membranes of different compartments. The role of two-pore calcium channels (TPC) for such signatures has been debated controversially, because evidence for localisation in both, the plasma membrane as well as in the tonoplast, has been proposed. Using a GFP fusion of the tobacco homologue *Nt*TPC1A in the background of tobacco BY-2 cells, we show that this channel is localised at the tonoplast. This localisation depends on actin filaments, but not on microtubules, as shown by pharmacological interference. Since the construct is driven by the constitutive Cauliflower Mosaic Virus 35S promoter, we can also detect phenotypic differences, such as impaired auxin-dependent cell elongation, reduced intracellular calcium content (that can be rescued by supplementation of calcium), and partial resistance to gadolinium, inhibitors of calcium influx. We also monitored the response to harpin, an elicitor from the phytopathogenic bacterium *Erwinia amylovora*. Here, the overexpressor line shows a higher sensitivity indicating that *Nt*TPC1Aparticipates in defence-related programmed cell death. The data are discussed with respect to a role of *Nt*TPC1A for spatial calcium signatures, and the regulation of cell growth by actin and auxin.

**Supplementary Information:**

The online version contains supplementary material available at 10.1007/s00709-025-02118-1.

## Introduction

Cells need to buffer their internal state against numerous external challenges. This holds, in particular, true for plant cells that are immobile and, therefore, cannot respond by moving to more favourable sites. To sustain homeostasis, a plant cell needs to perceive ambient fluctuations and to deploy a signal transduction culminating in adaptive responses able to restore the challenged equilibrium. Each challenge requires a specific adaptive response, which might be achieved by separate and parallel signal chains. However, at least for the early signals upstream of gene activation, different stress factors seem to make use of the same molecular events. This simple fact can lead to antagonistic (in case of competition for a limited signal component) or synergistic (in case of negative regulators) interactions, often summarised under the term cross-talk (for a conceptual review see Knight and Knight 2001).

A crucial signal, utilised by several signal chains, is calcium. The question, how the very same molecule can convey different content, depending on the signalling context, has led to the concept of calcium signatures, meaning that the information is conveyed by patterns of change rather than by the mere molecular nature of the signal. In fact, using plants expressing the luminescent calcium reporter aequorin, it was possible to demonstrate different temporal patterns for cytosolic calcium that differed specific for different stimuli, such as mechanic stimulation, cold stress, or bacterial attack (Knight et al. 1991). However, to demonstrate that the information is really encoded in these differential patterns as signature is far from trivial. Using a rhythmic change of buffers with high and low calcium, the calcium signature linked with the abscisic-acid guard-cell response could be restored in, *gca2*, a mutant of *Arabidopsis thaliana*, where the absence of a calcium-dependent kinase results in affected stomatal closure. The rhythmic incubation rescued the mutant phenotype, such that the stomata were able to close again (Allen et al*.*, 2001). Around a decade later, different calcium signatures could be imposed using a sequence of electrical pulses, and the plant response was then monitored by transcriptomics (Whalley and Knight 2013). Here, for each of the three imposed signatures (that were equivalent in terms of overall amplitude), a specific pattern of gene expression could be detected, providing evidence that it is indeed not the integrated abundance of cytosolic calcium, but its temporal pattern that is read out by the plant.

Calcium signatures are shaped by pumps that either allow influx of calcium into the cytoplasm either from the environment, or from intracellular stores, or remove calcium back to their original pool. The respective contribution of the individual channels depends on the respective environmental factor and can be quite different (for a recent review see Park and Shin 2022). Especially plasma membrane and tonoplast are endowed with a high number of different channels (recently reviewed in Blatt 2024) presumably allowing for a high variability of possible activity patterns.

For instance, several members of the cyclic nucleotide-gated channels (reviewed in Dietrich et al. 2020) located at the plasma membrane allow entry of calcium into the cytosol, supported by numerous channels with specific functions (for review see Blatt 2024). Calcium influx from internal stores contributes as well. An example is the release of calcium from the ER into the cytoplasm through the calcium/cation transporter (CCX2) that has been shown to be essential for salinity tolerance using loss-of-function and overexpression lines in *Arabidopsis* (Corso et al. 2018). The formation of a signature requires systems to remove calcium again—here, well-known systems are calcium ATPases and calcium proton exchangers (for review see Demidchik et al. 2018). Also, the role of organelles for calcium signatures has meanwhile attracted considerable attention (for a recent review see Resentini et al. 2021). The high complexity and apparent redundancy of calcium channels is certainly one factor contributing to the considerably controversy found in the field on function and regulation of these channels. A second factor may be that the different methodologies to study calcium channels, all come with their specific constraints, leading to discrepant conclusions—genetic ablation will give a static snapshot and is obscured by pleiotropy, localisation studies with fluorescent sensors have to deal with the problem that sensing of calcium will impact the level of free calcium, and patch-clamp studies require isolation of the membrane systems from their cellular context.

The complexity of calcium signalling along with these methodological limitations can even lead to situations, where the role of a given molecule as transporter of calcium can be discussed controversially. This is the case for the two-pore channels TPC1. The complex regulation of this channel that is, for instance, activated by its own cargo, calcium is reviewed in Hedrich et al. (2018). A seminal paper (Peiter et al. 2005) used patch-clamp of inside out mesophyll protoplasts patches of *Arabidopsis thaliana* to demonstrate a characteristic slow activation by calcium. The electrophysiological properties of this channel could be associated with the vacuole rather than with the plasma membrane. Therefore, TPC1 was originally discussed as vacuolar channel (for review see Pottosin and Schönknecht, 2007). However, attempts to infer its localisation by alternative approaches, casted doubt on the tonoplast model. By expressing a GFP-fusion in protoplasts, and by fractionation of vesicles, these authors tried to corrobate a vacuolar localisation. However, the images are rather showing a plasma membrane localisation, and to detect plasma membrane contaminations of tonoplast preparations is far from trivial. In fact, expression of TPC1-GFP fusions from cereals in the onion epidermis system, showed a clear plasma membrane localisation (Kurusu et al*.*, 2004; Wang et al. 2005). To test, whether this discrepancy might derive from a different subcellular localisation in dicots versus monocots, the *tpc1* mutant of Arabidopsis was complemented with the functional TPC1 from rice and the effect tested by patch-clamp of isolated vacuoles from the mutant and the complemented line (Dadacz-Narloch et al. 2013). The successful and specific complementation suggested that the rice TPC1 could rescue the vacuolar phenotype of the *Arabidopsis* recipient, providing evidence for a vacuolar localisation. However, as the other evidence proposed to demonstrate that TPC1 is localised at the tonoplast, this rescue represents rather indirect evidence.

Tobacco BY-2 cells represent an excellent system for cell biological studies, since they are relatively large, can be transformed with GFP-fused markers, and are not only vigorously proliferating, but also display axial cell expansion during the stationary phase of their culture cycle (Huang et al. 2017). These cells harbour two *Nt*TPC1 channels for their own (Kadota et al. 2004) that were able to complement a yeast mutant defective in a calcium channel. By overexpression of *At*TPC1 in these cells, these authors observed a co-suppression of the endogenous channels and used this as proxy to analyse a loss-of-function phenotype, making use of aequorin as calcium reporter. They could show that cytosolic calcium accumulation in response to a fungal elicitor or to osmotic challenges required the activity of those channels. The subcellular localisation of these channels was not addressed, however.

So far, to our knowledge, a convincing demonstration that *Nt*TPC1A is localised in the tonoplast of intact cells, is still missing. We ventured, therefore, to generate a GFP-fusion of this transporter and test its subcellular localisation in tobacco BY-2 cells as experimental model well suited for cell biological studies.

## Material and methods

Generation of tobacco BY-2 lines overexpressing *Nt*TPC1A as GFP fusion. RNA was extracted from proliferating tobacco BY-2 (*Nicotiana tabacum* L. cv. ‘Bright Yellow 2’) suspension cells at day 3 after subcultivation. Cells were collected by centrifugation at 4000 rpm for 2 min in a 2-ml reaction tube (Eppendorf). After draining off the culture medium, cells were immediately frozen in liquid nitrogen and homogenised to a powder (TissueLyser, Qiagen, Hilden, Germany). Total RNA was extracted with the innuPREP Plant RNA kit (Analytik, Jena) according to the protocol of the manufacturer, followed by digestion of genomic DNA digested on column with RNase-free DNAse I (Qiagen) for 30 min at 30 °C. The eluted RNA was tested for integrity by agarose gel electrophoresis and purity by spectrophotometry (Nanodrop). A template of 1 µg of RNA was reversely transcribed using the DyNAmo DNA Synthesis Kit (Finnzymes, Vantaa, Finland) following the protocol of the producer. Full-length Nt*Nt*TPC1A (GenBank AB12464 6.1) was cloned using a PCR-based GATEWAY strategy employing the primer pair *Nt*TPC1A-fw and *Nt*TPC1A-rev (details are given in Suppl. Table S1). The size of the amplicons was verified by electrophoresis and purified (NucleoSpin, Macherey–Nagel, Düren, Germany) according to the instructions of the manufacturer. Based on the specific motifs of the primers, these amplicons could be integrated into the GATEWAY entry vector and from there, based on recombination into the target vector pK7FWG2,0 (Karimi et al. 2002) driving expression of a C-terminal fusion with GFP driven by the constitutive CaMV-35S promoter. Tobacco BY-2 cell lines, expressing this construct in a stable manner were generated following the protocol by Klotz and Nick (2012) using electroporation (2.5 kV, 200 Ω, 5 ms, Gene Pulser Xcell™, Bio-Rad, München, Germany) of A. *tumefaciens* strain LBA 4404 (Invitrogen, Paisley, UK). Co-cultivation was conducted at 22 °C, screening of transgenic calli at 26 °C.

Phylogenetic analysis of *Nt*TPC1A. The cDNA sequence obtained for *Nt*TPC1A was converted into the corresponding amino-acid sequence using the SwissProt Translate Tool (https://web.expasy.org/translate/). Homologues across the different classes of Streptophytes were searched based on the peptide sequence using the BLAST option of the Expasy database (web.expasy.org) and aligned in MEGA-X (www.megasoftware.net, Kumar et al. 2018) using the MUSCLE algorithm. Since *Nt*TPC1A is a very complex protein, likely originated by merging of different, previously unlinked domains, we inferred the tree not by Maximum Likelihood, but by the more robust Neighbour Joining algorithm (Saitou and Nei 1987), because it performs better in such cases (for a detailed treatment see Yoshida and Nei 2016). The tree over the 1024 positions of the alignment was then tested for bootstraps using 500 replications (Felsenstein 1985), collapsing branches with bootstrap values below 50%. The tree was drawn to according to distance based on Zuckerkandl and Pauling (1965).

Cell culture and drug treatment. The cells were maintained in suspension in 3% w/v sucrose in a modified Murashige-Skoog medium (4.3 g^.^L^−1^ Murashige and Skoog salts, Duchefa Biochemie, Haarlem, The Netherlands) supplemented with 200 mg^.^L^−1^ KH_2_PO_4_, 100 mg^.^L^−1^ inositol, 1 m mg^.^L^−1^ thiamine, and 0.2 m mg^.^L^−1^ 2,4 dichlorophenoxyacetic acid, at pH 5.8 subculturing every 7 d by inoculation of 0.5 g fresh weight per 30 mL of fresh medium in 100-mL Erlenmeyer flasks. It should be mentioned that the Murashige-Skoog medium contained 0.32 g^.^L^−1^ CaCl_2_ (corresponding to 2.88 mM). To sustain selective stringency for expression of the transgene, 100 µg^.^mL^−1^ Kanamycin were added to the medium. Cells grew under continuous shaking at 150 rpm on an orbital shaker (IKA Labortechnik, Staufen, Germany) at 26 °C in the dark. rotating constantly at 150 rpm. As backup, cells were plated on the same medium solidified by 0.8% (w/v) Phytoagar (Roth, Karlsruhe, Germany) at monthly passages. Harpin, an elicitor of the phytopathogenic bacterium *Erwinia amylovora*, can induce a severe remodelling of the actin cytoskeleton followed by programmed cell death (Chang et al. 2015) and was used in some experiments at a concentration of 27 µg^.^mL^−1^ diluted from a commercial biocontrol agent (Messenger, EDEN Bioscience Corporation, Washington, USA; 3% of active ingredient Harpin protein) stored as a 300 mg^.^mL^−1^ stock solution (equivalent to 9 mg^.^mL^−1^ of harpin protein). Latrunculin B and Phalloidin were used at 2 µM, into the cell suspension, Oryzalin and Taxol at 20 µM. All cytoskeletal drugs were diluted into the cell suspension from 1 mM stock solutions in DMSO that were stored at 4 °C. The auxin indole-3-acetic acid (IAA) and the auxin-transport inhibitor 1-N-naphthylphthalamic acid (NPA) were diluted from an ethanolic stock solution of 10 mg^.^mL^−1^. All compounds were purchased Sigma-Aldrich (Deisenhofen, Germany).

Colocalisation analysis of Nt*Nt*TPC1A with actin and the plasma membrane. To follow the spatial relation between Nt*Nt*TPC1A and actin filaments, the tobacco BY-2 cells expressing *Nt*TPC1A-GFP were transiently transformed with a RFP fusion of the actin-binding domain of plant fimbrin in the GATEWAY vector p2RGW7 (Maisch et al. 2009). Gold particles (120 mg, diameter 1.5–3.0 µm; Sigma–Aldrich, Deisenhofen) were suspended in 1 ml 50% (v/v) sterile glycerol by mixing on a platform vortexer (Bender & Hobein, Zurich, Switzerland). Aliquots of 12.5 µL of gold suspension were transferred to a reaction tube. While mixing vigorously on the vortexer, the following components were added successively: the dissolved DNA (1 µg), 12.5 µL of 2.5 M sterile CaCl_2_, and 5 µL of 0.1 M sterile spermidine (Roth, Karlsruhe, Germany). Continuous vortexing is mandatory to ensure homogeneity of coating and needs to be continued for additional 3 min. Then, the DNA coated gold particles were spun down briefly, and the supernatant was discarded. Subsequently, the gold particles were washed with 125 µL of ice-cold absolute ethanol and resuspended in 40 µL of ice-cold absolute ethanol. Next, the DNA coated gold particles were loaded onto the macrocarrier (BioRad, Hercules, CA, USA) in 10 µL steps. Particle bombardment was performed immediately after complete evaporation of the ethanol.). Aliquots of 800 µL BY-2 cell suspension, collected at day 3 after subcultivation were allowed to settle for 5 min in a reaction tube before draining 300 µL of the supernatant. The remaining 500 µL of cells were then resuspended in the residual medium and evently spread on 1.5 mL solidified culture medium in Petri Slides TM (Millipore, Billerica, USA). Cells were bombarded three times at a pressure of 1.5 bar in a vacuum chamber at −0.8 bar in a custom-build biolistic device (Finer et al. 1992). After the bombardment, the cells were allowed to express the transgene for 16 h at 26 °C in the dark before microscopic analysis. To address the localisation of *Nt*TPC1A in relation to the plasma membrane, *Nt*TPC1A-GFP cells were stained with 5 µM of the polystyryl dye FM4-64 (Molecular Probes/Invitrogen, Carlsbad, CA, USA). The labelling of the plasma membrane is saturated after 60 s and, subsequently, the label already moves on to endosomes (Liu et al. 2013). Therefore, cells were mounted for microscopy directly after adding the dye. To quantify cross-localisation of *Nt*TPC1A-GFP to the plasma membrane (labelled by FM4-64), an ImageJ based protocol was used, where the images were first split into the channels and then histograms across tonoplast and plasma membrane were collected in the red and the green channel using a spline-fitted line width of 8 px to reduce noise from local inhomogeneities. Peak heights from the intensity profiles along the probing line for the two channels were used to determine channel cross-bleed and, resulting from that, the cross-localisations of the *Nt*TPC1A-GFP and the FM4-64 signals as outlined in Suppl. Fig. S6. Data represent mean and standard error from 12 individual cells.

Expression analysis. Steady-state transcript levels for Nt*Nt*TPC1A were determined by RT-qPCR from mRNA extracted at day 4 after subcultivation (at the onset of cell expansion) using the innuPREP Plant RNA kit (Analytic, Jena) with column digestion of genomic with RNase free DNAse I (Qiagen, Hilden) for 30 min. Cells were drained from supernatant, shock frozen in liquid nitrogen and homogenised to a poweder (TissueLyser, Qiagen, Hilden). Quality and integrity of the purified RNA were controlled by spectrophotometry (NanoDrop) and agarose gel electrophoresis. A template of 1 µg total RNA was reversely transcribed into cDNA employing the DyNAmo TM cDNA synthesis kit (Thermo Fisher Scientific Inc, Waltham, MA, USA) following the instructions of the manufacturer. To amplify a specific fragment of Nt*Nt*TPC1A, the primer qNt*Nt*TPC1A-fw and qNt*Nt*TPC1A-rev (details are given in Suppl. Table S1) were used. Based on preparatory studies, L25 and GAPDH were selected as internal standards, because their expression was found to be stable. The respective primer pairs are given in Suppl. Table S1. The specificity of the amplification was analysed by inspecting the melting curve analysis. Amplification was carried out in 20-μl reactions 200 nM of each primer, 200 nM of each dNTP, 1xGoTaq colourless buffer, 2.5 mM supplemental MgCl_2_, 0.5 u GoTaq polymerase (Promega, Mannheim, Germany), 1xSYBR green I (Invitrogen, Darmstadt, Germany), and 1 µl of a 1:10 cDNA dilution. Data represent four biological replicates in technical triplicates. The relative expression level of each gene was calculated with the ΔC_t_ method (Livak and Schmittgen 2001) using the L25 and GAPDH for normalisation.

Confocal microscopy. Cells were phenotyped with help of an AxioImager Z.1 microscope (Zeiss, Jena, Germany) equipped with an ApoTome microscope slider for optical sectioning and a cooled digital CCD camera (AxioCam MRm; Zeiss). The GFP signals were observed through the filter set 38 HE (excitation, 470 nm; beamsplitter, 495 nm; and emission: 525 nm) respectively (Zeiss). To quantify cell mortality and cell morphology, samples were observed by Differential Interference Contrast (DIC) using a 20 × objective (Plan Apochromat 20x/0.75). Symmetric sampling was ensured by the MosaiX module of the imaging software (Zeiss). Images were processed and analysed using the AxioVision (Rel. 4.8.2) software. To collect details on subcellular localisation of the GFP signals reporting *Nt*TPC1A, we conducted spinning disc confocal microscopy using an AxioObserver Z1 microscope equipped with a laser dual spinning disk scan head from Yokogawa (Yokogawa CSU X1 Spinning Disk Unit, Yokogawa Electric Corporation, Tokyo, Japan), and a cooled digital CCD camera (AxioCam MRm; Zeiss). GFP was excited with the 488-nm line, RFP and FM4-64 with the 564-nm line of an ArKr laser through a Plan Apochromat 63x/1.44 DIC oil objective operated via the Zen 2012 (Blue edition) software platform. Acquired images were processed with respect to size, contrast, and brightness by Photoshop (Adobe Systems, San Jose, CA, USA).

Calcium measurements. Intracellular calcium content was measured by Atomic Absorption Spectrometry (AAS) in expanding cells (sampled at day 5 after subcultivation). Cells were harvested by filtration through filter paper in a Büchner funnel under a mild vacuum (500 Pa, Vacuubrand CVC2, Brand, Germany) and then washed with a tenfold volume of Millipore water. After draining the water by vacuum filtration, the cells were dried at 80 °C for 3 days. Aliquots of 150 mg were digested with 5 ml of 65% nitric acid (Roth, Karlsruhe, Germany) in 10-ml digestion tubes (Gerhardt, UK) at 20 °C overnight, followed by boiling for 2 h in a water bath. After cooling, 4 ml of the digestion product were mixed with 21 ml of millipore water and subjected to AAS (AAnalyst 200, Perkin Elmer). The contents of sodium and calcium were measured in an air acetylene flame and referred to dry weight. Data points represent mean and standard error from at least 3 independent experimental series.

Cellular phenotyping. For the determination of cell division synchrony, aliquots of 500 µL cell suspensions were sampled from days 1 to 4 after subcultivation and investigated, upon appropriate dilution with culture medium, by Differential Interference Contrast (AxioImager Z.1, Zeiss) from images obtained from differential interference contrast (DIC) by a digital imaging system (AxioVision, Zeiss), using the MosaiX module to cover a 4 × 4 mm area from 121 individual frames. Frequency distributions were constructed over the number of cells per individual files. Each data point represents at least 1500 cell files from three independent experimental series. Cell width and cell length at subcultivation were measured in the central section of the cells using the perimeter function of the AxioVision software. Again, each data point represents mean and standard error from at least 1500 individual cells obtained from three independent experimental series. Cell mortality was determined by the Evans Blue Dye Exclusion test (Gaff and Okong’O-Ogola 1971) with minor modifications as described in Wang et al. (2022) using at least 33,000 cells from at least least three independent experimental series. Growth was estimated by measuring the packed cell volume (PCV) volume at day 5 after subcultivation as described in Kühn et al. (2013). Here, each data point represents mean and standard error from at least three independent experimental series.

## Results

### Tobacco BY-2 cells express a canonical member of the TPC1a family

**Fig. 1:**
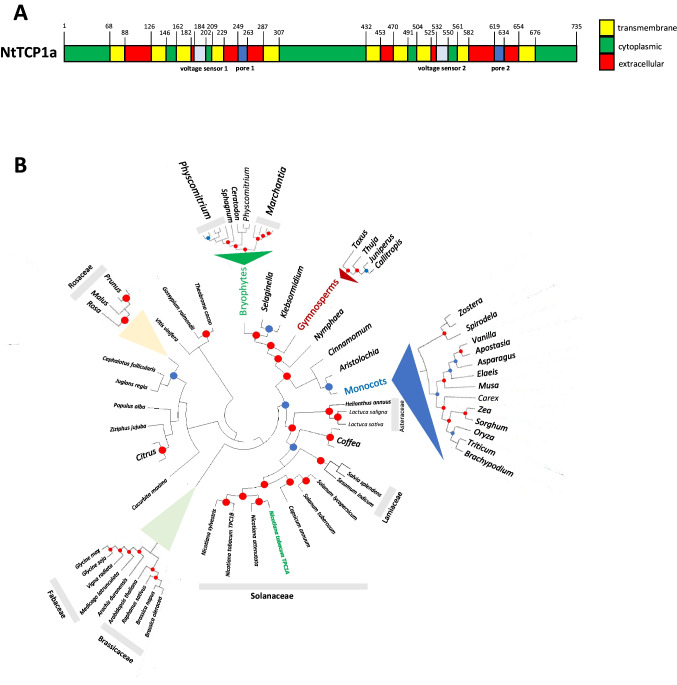
Domain structure (**A**) and phylogenetic relationship (**B**) of the Two Pore Channel 1 (*Nt*TPC1A) isolated from tobacco BY-2 cells. Blue circles indicate bootstrap values above 50%, red circles represent for bootstrap values of > 99%

The study was conducted in tobacco BY-2 cells as cellular model. Therefore, *Nt*TPC1A was cloned and sequenced from this model system by a RT-PCR-based strategy from transcripts of BY-2 cells. The amino-acid sequence inferred from the full-length cDNA sequence was analysed for the presence of the domains and motifs required for a function as a two-pore channel protein. The sequence contained the characteristic ten transmembrane domains, the two voltage sensor motifs, as well as the two pore domains and, thus, harbours all the features characteristic for this type of channel protein (Fig. 1A). A phylogenetic analysis (Fig. 1B) placed this protein central into one clade with other members of the TPC1 family from the Solanaceae. While this phylogeny is compatible with the tree shown in Hedrich et al. (2018), which covers a wider evolutionary distance, there are a couple of details that are highlighted: Sister clades comprised homologues from the Lamiaceaa and Asteraceae and, thus, more or less, reflected the evolutionary relationship. This is not trivial, since other Angiosperm homologues, for instance from the Rosaceae, or the Brassicaceae and Lamiaceae, were fairly distant, comparable to the distance with the clade comprising the monocots and basal Angiosperms. This aspect might be relevant, when conclusions obtained in Arabidopsis are to be transferred to other taxa. A couple of more distantly related homologues could be recovered from gymnosperms, lower land plants, and Streptophytes, but, here, their topology was consistent with the evolutionary distance. A few Angiosperm homologues (*Vitis*, and the representatives of the Malvales, *Gossypium* and *Theobroma*) diverged considerably leading to the question, whether these taxa harbour a true homologue at all.

Overall, both by the protein structure, as well as by phylogenetic relationship, the cloned tobacco *Nt*TPC1A can be considered as a canonical member of the two-pore channel family.

### Overexpression of tobacco *Nt*TPC1A depletes the pool of intracellular calcium

**Fig. 2:**
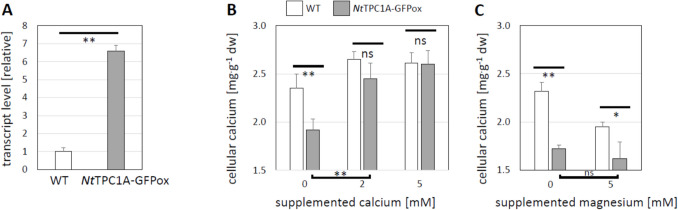
Overexpression of native tobacco *Nt*TPC1A as fusion with GFP in tobacco BY-2 cells. **A** Steady-state transcript levels in non-transformed tobacco BY-2 cells (WT) as compared to the *Nt*TPC1A-GFP overexpressor (*Nt*TPC1A-GFPox) **B** Intracellular calcium contents in both cell strains prior to (0) and after supplementation with external calcium **C** Intracellular calcium contents in both cell strains without and with supplementation with external magnesium as alternative divalent cation. Significance of differences was probed by a Student *t*-test at *P* < 0.05 (*) or *P* < 0.01 (**), ns not significant at *P* = 0.05. Data represent mean values and standard errors from three independent experimental series. Transcript abundance was measured in technical triplicates for each biological replication

We generated a tobacco BY-2 cell strain overexpressing the innate tobacco *Nt*TPC1A in fusion with GFP. Steady-state transcript levels were around 6.5-fold elevated over those seen in the non-transformed wild type (Fig. 2A). When we measured the content of intracellular calcium (Fig. 2B), we found them to be reduced to half of those seen in the wild type. However, this phenotype could be rescued by adding calcium to the medium. Already for 2 mM of supplemented calcium (corresponding to 5.88 mM Ca^2+^ in total, since the medium contained 2.88 mM CaCl_2_), the intracellular calcium content had approximated those seen in the wild type to a degree that the difference was not any longer significant. For 5 mM of supplemented calcium (corresponding to 7.88 mM of total Ca^2+^), the values became even identical. This rescue is specific for calcium and was not achieved by magnesium as alternative divalent cation (Fig. 2C).

### Tobacco *Nt*TPC1A-GFP is localised to the tonoplast throughout the cell cycle

**Fig. 3:**
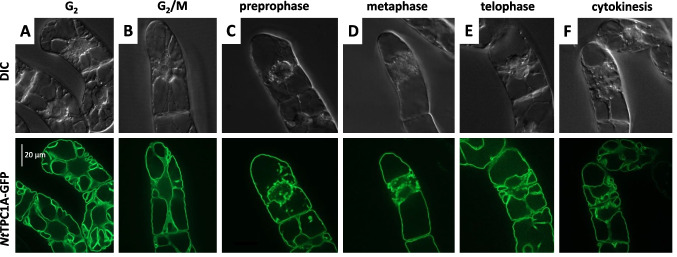
Subcellular localisation of *Nt*TPC1A-GFP during the cell cycle. Representative cells of the respective phase are represented in both, Differential Interference Contrast (DIC) to show the structure of the cytoplasm, as well as for the GFP signal reporting the localisation of tobacco *Nt*TPC1A. Images show confocal sections through the cell centre

We followed the localisation of *Nt*TPC1A as reported by the fused GFP by means of spinning-disc confocal microscopy (Fig. 3). Depending on the phase of the culture cycle, the division activity differs. The peak is at around day 3 after subcultivation with more than 3% of cells in mitosis and a correspondingly high fraction of cells in G_2_ reached even without synchronisation (Huang et al. 2017). At this time point, it is fairly convenient to locate cells in different stages of the cell cycle. In G_2_, when the nucleus had moved to the cell centre, the GFP signal lined the transvacuolar strands, but was also seen, in a wavy line, adjacent to the plasma membrane (Fig. 3A). At the transition of G_2_ to the M-phase, the cytoplasm organised into the characteristic Maltesian cross, which was truly reflected in the GFP signal, lining the cytoplasmic strands, while the signal underneath the plasma membrane persisted (Fig. 3B). During pre-prophase, when the cytoplasm reorganised, such that the vacuole separated into the two vacuoles of the prospective daughter cells, the GFP signal followed the remodelling of the tonoplast (Fig. 3C), and this remained so during metaphase as well (Fig. 3D). When, in telophase, the daughter nuclei emerged accompanied by the appearance of new cytoplasmic strands, these were again lined with the GFP signal (Fig. 3E). During the subsequent cytokinesis, the vacuolar systems of the daughter cells became completely separated and this was again reflected in a corresponding remodelling of the GFP signal (Fig. 3F).

**Fig. 4:**
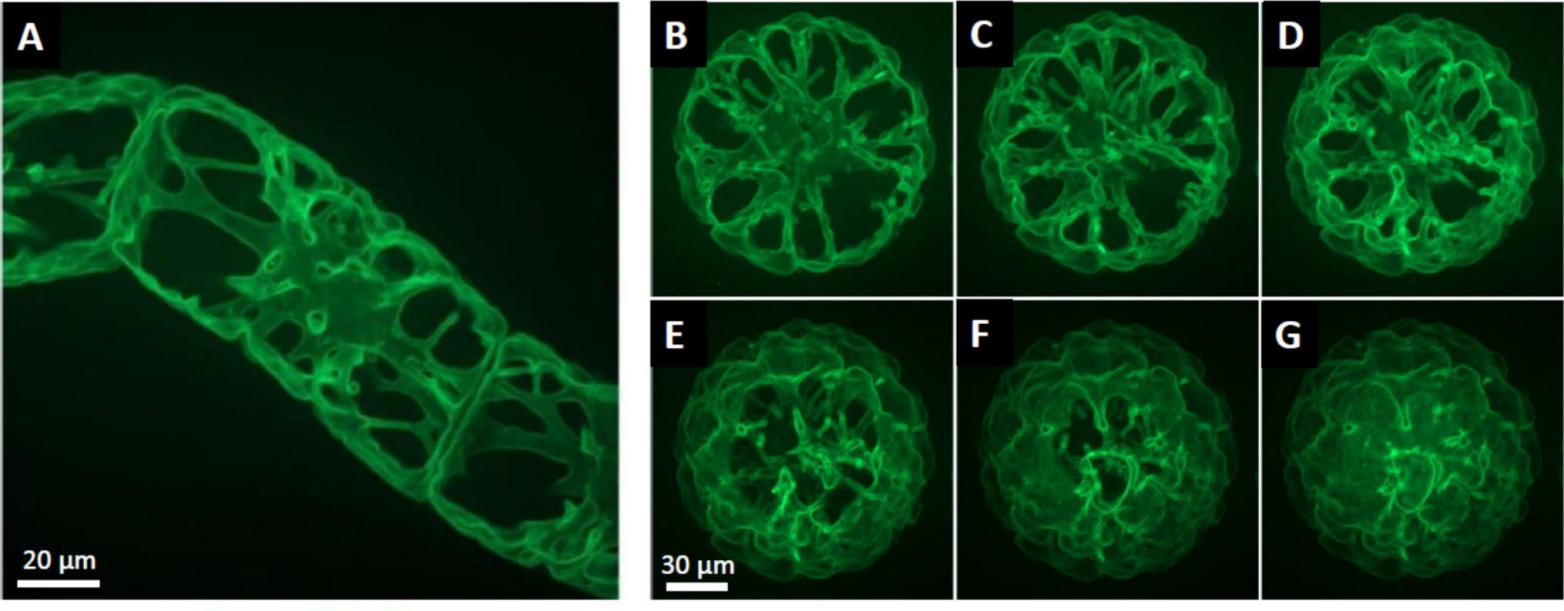
Subcellular localisation of *Nt*TPC1A-GFP in a walled interphase cell (**A**) and in a protoplast derived thereof (**B–G**). Geometric projection of a confocal z-stack across the central part of the cell is shown in **A** to give an impression of the three-dimensional structure. For the protoplast (**B–G**) individual sections of a z-stack recorded from the cell centre (**B**) to the cell periphery (**G**) are shown to visualise that the signal is found in the tonoplast, but not in the plasma membrane

To visualise the three-dimensional organisation of the GFP signal, confocal z-stacks were collected across the central part interphase cells and subjected to geometrical projection (Fig. 4A). This produced a clear, contiguous line with the tonoplast and visualised the complex topology of the vacuole. The cytoplasmic strands were only faintly fluorescent from the tonoplast sections above and below. Instead, the vacuole appeared in black because it reached beyond the borders of the stack. To test, whether the signal is also found in the plasma membrane, protoplasts were generated, and confocal stacks collected across them, starting from the cell centre (Fig. 4B) and ending close to the periphery (Fig. 4G). These sections allowed to follow the topology of the vacuole with chambers and lacunae separated by cytoplasmic strands. Towards the periphery these structures were of a wavy appearance and by no means, the strictly spherical line pattern was seen, as it would be expected for a protein localised to the plasma membrane (Fig. 4E-G). Thus, the GFP signal reporting *Nt*TPC1A, is exclusively localised to the tonoplast. To corrobate this conclusion, we also conducted a double visualisation with the polystyryl dye FM4-64 (Suppl. Fig. S1). This dye is rapidly inserted into the plasma membrane, but soon after passed on to endosomes, and eventually even reaches the tonoplast. To minimise cross-labelling of FM4-64 to the tonoplast, the cells were viewed rapidly (at around 1 min after addition to the dye). Intensity profiles were collected along probing lines orthogonal to plasma membrane and tonoplast and the heights of the intensity peaks were used to determine channel cross-bleeding. Based on the cross-bleeding, the cross-localisation of the respective signal could be estimated. Here, the putative *Nt*TPC1A signal at the plasma membrane was found to be around 5% of the signal found in the tonoplast. In contrast, a significantly higher proportion of the FM4-64 signal had already reached the tonoplast (around a quarter of the signal found in the plasma membrane.

### Overexpression of *Nt*TPC1A-GFP impairs cell elongation

**Fig. 5:**
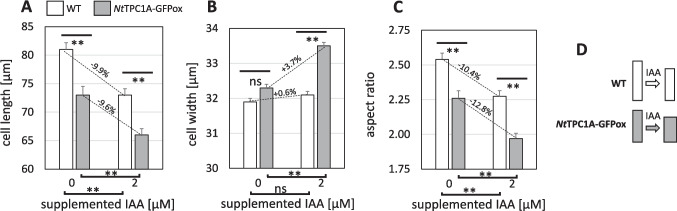
Phenotype of cells overexpressing *Nt*TPC1A-GFP (*Nt*TPC1A-GFPox, grey bars) as compared to non-transformed tobacco BY-2 cells (WT, white bars) as assessed at the end of one cultivation cycle (7 days) without or with 2 µM of supplemented indole acetic acid (IAA). **A** Cell length. **B** Cell width. **C** Aspect ratio. **D** Schematic visualisation of the shape change in response to IAA. Data points represent mean and standard error from at least 1500 individual cells obtained from three independent experimental series. Statistical significance of differences was tested by a Student *t*-test with ns non-significant, and ** significant at *P* < 0.01

To get insight into potential cellular functions of *Nt*TPC1A, we phenotyped the overexpressor line as compared to the non-transformed wild type. The morphology of the overexpressor cells was significantly different (Fig. 5)—they were shorter and wider, such that their aspect ratio was lower. When the wild type was cultivated in presence of 2 µM indole acetic acid (IAA), cells were shortened to the value seen in the overexpressor without auxin (Fig. 5A). However, in the overexpressor, this supplemental auxin caused an even further reduction in length. In contrast, supplemental IAA made the cells of the overexpressor significantly wider, while the cells of the WT maintained their original width (Fig. 5B). As a result of the reduced elongation, which in relative terms was comparable, both cells responded to IAA with a reduction of aspect ratio (Fig. 5C). However, the widening observed in the overexpression line rendered this decrease of aspect ratio more pronounced as compared to the wild type.

### Localisation of *Nt*TPC1A-GFP depends on actin filaments, but not on microtubules

**Fig. 6:**
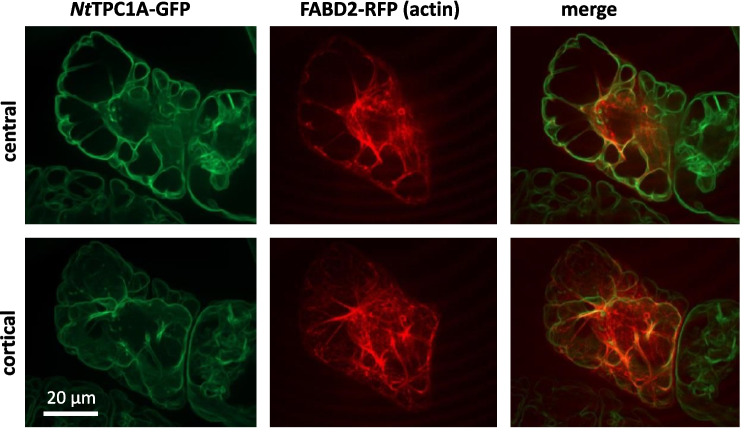
Co-localisation of *Nt*TPC1A-GFP with the actin marker fimbrin actin-binding domain 2 (FABD2)-RFP transfected transiently into the background of the *Nt*TPC1A-GFP line. Representative confocal sections collected in the cell centre, or the cell cortex are shown

Since *Nt*TPC1A-GFP lined cytoplasmic strands and also displayed a Maltesian cross pattern at the G_2_-M transition (Fig. 3), we reckoned, whether it might interact with actin filaments. A further reason was the impact of NTTPC1 overexpression on the response to transportable auxin and to the auxin-efflux inhibitor NPA. (Fig. 5, Suppl. Fig. S2). To visualise actin, we transiently transfected the fimbrin actin-binding domain 2 in fusion with RFP into the background of the *Nt*TPC1A-GFP line (Fig. 6). We observed a close colocalisation of perinuclear actin cables tethering the nucleus in the cell centre during G_2_, while the GFP signal in the cell periphery was less intensely labelled, indicating that the cortical actin meshwork is not associated with *Nt*TPC1A-GFP.

**Fig. 7:**
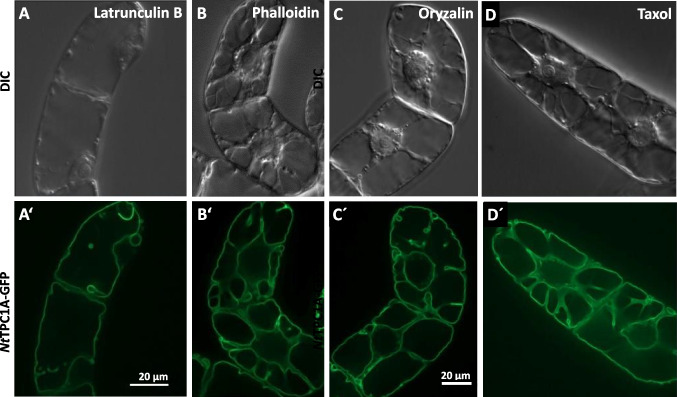
Response of *Nt*TPC1A-GFP to modulation of actin filaments (**A**, **A’**–**B**, **B’**) and microtubules (**C**, **C’**–**D**, **D’**). Representative cells after treatment with 1 µM of Latrunculin B, eliminating actin filaments (**A, A’**) or 1 µM of Phalloidin stabilising actin filaments (**B**, **B’**), after treatment with 20 µM of Oryzalin, eliminating microtubules (**C**, **C’**) or 20 µM of Taxol stabilising microtubules (**D**, **D’**). Geometric projection of a confocal z-stack across the central part of the cell are shown in **A’**–**D’**, while the same cells in Differential Interference Contrast (DIC) are shown in **A**–**D**

A colocalisation indicates that *Nt*TPC1A-GFP is tethered to actin, but it is by no means proof for a causal link. Therefore, we tested whether the subcellular localisation of *Nt*TPC1A-GFP is impacted, when actin filaments are eliminated by Latrunculin B. This is, in fact, the case (Fig. 7A’). The fluorescent strands transversing the vacuole and lining the nuclear envelope, characteristic for cells in G_2_ (Fig. 6), disappear, and the signal is mostly seen lining the plasma membrane, with only few lacunae protruding into the cell interior. In the Differential Interference Contrast (Fig. 7A), one can observe that the transvacuolar strands have disappeared, and the nucleus has shifted to the cell periphery. When actin filaments are stabilised by Phalloidin (Fig. 7B, B’), both the transvacuolar strands as well as the reticulate pattern of *Nt*TPC1A-GFP transversing the vacuole are retained, as well as the central positioning of the nucleus.

To test, whether the changed localisation is specific for elimination of actin filaments, or whether it is a general consequence of an impacted cytoskeleton, we conducted control experiments, where microtubules were either eliminated (Fig. 7C, C) or stabilised (Fig. 7D, D’). Here, we did not observe any change in the localisation of *Nt*TPC1A-GFP. However, unlike as for Latrunculin B, we also did not observe a breakdown of transvacuolar cytoplasmic strands or a loss of central nuclear positioning. Thus, the localisation of *Nt*TPC1A depends on the architecture of transvacuolar cytoplasmic strands, and this architecture requires an intact actin cytoskeleton, while microtubules (at least at these time points) seemed dispensable.

### Overexpression of *Nt*TPC1A-GFP modulates response to calcium-channel blockers

**Fig. 8:**
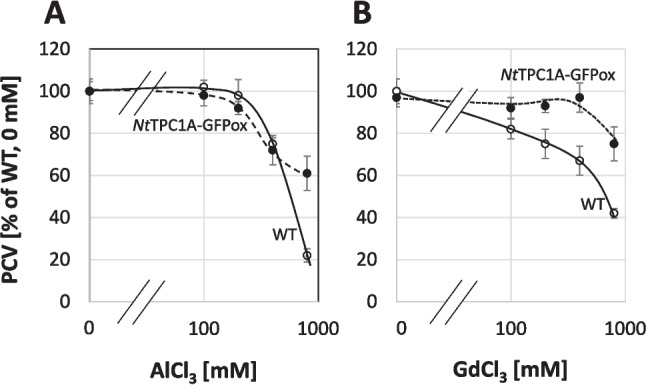
Dose–response relation for cell growth over the concentration of A aluminium chloride and B gadolinium chloride supplemented to the medium in non-transformed tobacco BY-2 cells (WT) and cells overexpressing *Nt*TPC1A-GFP (*Nt*TPC1A-GFPox). Growth is given as Packed Cell Volume relative to the non-transformed wild type grown under control conditions measured at the end of the culture cycle, at day 7. Gadolinium ions block calcium influx channels, aluminium was used as alternative three-valent cation without a specific effect on calcium channels. Data represent mean and standard error from three independent experimental series

Since the *Nt*TPC1A-GFP overexpressor line exhibited intracellular calcium depletion which could be rescued by exogenous calcium (Fig. 2B), we tested, whether the cellular response to Gadolinium known to inhibit calcium influx would be modulated upon overexpression of *Nt*TPC1A-GFP. Monitoring Packed Cell Volume as readout (Fig. 8B), we observed that in non-transformed cells growth became inhibited from 100 µM and dropped to 40% at 800 µM. For the overexpressor line, growth was not affected till 400 µM and for the highest tested concentration, 800 µM was still almost 80% of the control level, meaning that the threshold of inhibition was shifted by almost an order of magnitude upon overexpression of *Nt*TPC1A-GFP. To test, whether this effect was specific for Gadolinium, we conducted a control experiment with Aluminium as alternative three-valent cation (Fig. 8A) reported to block cytosolic calcium release (Kawano et al. 2004). Here, the inhibition of wild type and *Nt*TPC1A-GFP overexpressor were similar. Only at the highest concentration of aluminium, 800 µM, the transgenic line performed better than the non-transformed wild type.

### Overexpression of *Nt*TPC1A-GFP leads to a mild mitigation of salinity-induced toxicity

Calcium influx represents one of the earliest responses to both biotic, and abiotic stresses. A comparative study between two grapevine cell lines differing with respect to their salinity tolerance (Ismail et al. 2014) had shown that the swiftness and amplitude of early calcium influx correlated with tolerance. This prompted us to test, whether the *Nt*TPC1A-GFP overexpressor line would be more tolerant to cold stress. A dose–response relation of Packed Cell Volume over the concentration of sodium in the medium (Suppl. Fig. S3A) followed a sigmoidal curve that was identical between the non-transformed cells of the wild type and the overexpressor. However, for salt-induced mortality, there was a significant shift for the overexpressor towards higher concentrations of NaCl. To reach the mortality seen in the wild type at a given concentration of salt, the overexpressor required around 20 mM higher concentrations, meaning that the elevated expression of *Nt*TPC1A mitigated the mortality in response to salinity.

### Overexpression of *Nt*TPC1A-GFP promotes defence-related cell death

**Fig. 9:**
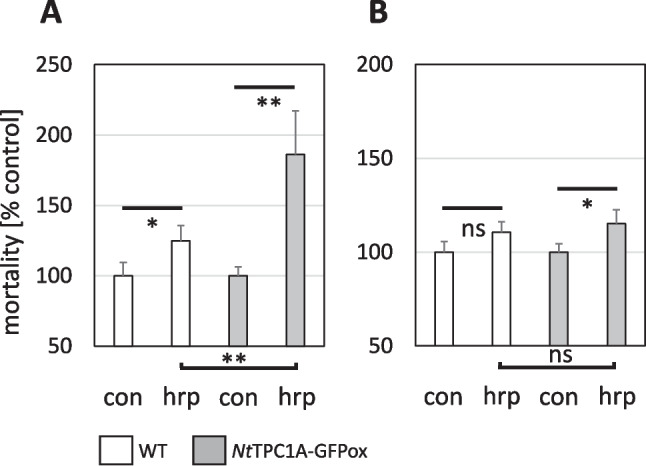
Response of cellular mortality to 9 mg^.^mL^−1^ of the bacterial elicitor harpin scored at 24 h after elicitation either at day 3 (**A**) or at day 5 (**B**) after subcultivation. Data represent mean and standard deviations from three independent experimental series comprising a population of 1500 cells. Statistical significance of differences was tested by a Student *t*-test with ns non-significant, * significant at *P* < 0.05, and ** significant at *P* < 0.01

To probe for a potential role of *Nt*TPC1A in the response to biotic stress, we used the bacterial elicitor harpin that can induce a defence-related cell death mimicking a pathogen-induced hypersensitive response. Since programmed cell death depends on the cell cycle, we conducted the experiment at two phases of the culture cycle, at day 3 after subcultivation, cells are cycling and preferentially in G_2_, while at day 5 after subcultivation, they are elongating and predominantly in G_1_. Since the ground levels of mortality were slightly different between the cell lines and also between the culture phases, we plotted the results relative to the mortality seen in the non-transformed and non-treated wild type. Ground mortalities were 8.4% for the wild type and 11.5% for the *Nt*TPC1A overexpressor at day 3. For day 5, the values were 7.4% for the wild type, and 9.9% for the overexpressor. Elicitation by harpin increased mortality in the wild type by 25% at day 3 (Fig. 8A), which was significant, for day 5, the increase was around 10% (Fig. 8B) and did not cross the threshold for significance. For the overexpression, mortality in response to harpin was more substantial with around 80% at day 3 and 20% at day 5. Thus, independently of genotype, the responsiveness to harpin was generally higher at day 3, when the majority of cells was in G_2_. The response in the *Nt*TPC1Aoverexpressor was more pronounced as compared to the non-transformed wild type (Fig. 9). This amplification was most pronounced for day 3, but still detectable for day 5, despite the much lower amplitude in those cells that were mostly in G_1_.

### *Nt*TPC1A-GFP allows to follow vacuolar remodelling in response to harpin

**Fig. 10:**
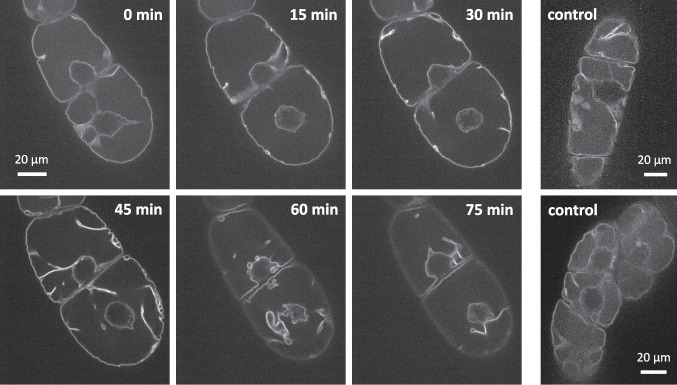
Vacuolar remodelling in response to 9 mg.mL^−1^ of the bacterial elicitor harpin in representative cells treated at day 5 after subcultivation, in the expansion phase. Images show frames from a representative time-lapse series confocal sections collected in the nuclear plane

As to get insight into early events of harpin-induced vacuolar remodelling, we used the *Nt*TPC1A-GFP line as tonoplast marker, following the tonoplast response in individual cells over time. The experiment was conducted at day 5 after subcultivation, when most cells had stopped cycling and had passed through the transition towards cell expansion. In those cells, a central vacuole was already fully developed and the GFP signal lined the transvacuolar cytoplasmic strands converging on the cytoplasmic pocket harbouring the nucleus. Adjacent to the cell border, the signal was found in a contiguous, but wavy line as to be expected from a tonoplast signal in those cells, where the peripheral cytoplasm is confined to a very thin rim underneath the plasma membrane. In response to harpin, we observed from around 15 min that the signals became more intense and also differed between subsequent frames, indicating movement of transvacuolar strands (Fig. 10). From around 60 min, the signal lost linearity and formed circles and loops, also around the nucleus hinting towards a breakdown of the vacuole. Later, zig-zagging fluorescent lines could be observed that failed to delineate compartments.

As control, we also investigated the response of cycling cells, treated at day 3 after subcultivation (Suppl. Movie S4, Suppl. Fig. S5). Here, we observed that harpin induced a rapid and extensive remodelling of the tonoplast within minutes after elicitation. In contrast, the tonoplast remained stable in unchallenged control cells.

## Discussion

As to resolve the controversial record on the subcellular localisation of the two-pore calcium channel *Nt*TPC1A, we generated a transgenic tobacco cell line expressing a GFP fusion of the tobacco homologue *Nt*TPC1A and found this channel clearly localised at the tonoplast. While the subcellular localisation is certainly the central finding of this work, the overexpression of *Nt*TPC1A allows to extract information on the cellular functions of this channel adding to previous work on NTTPC1 that was either done in patch-clamp models (perfect for biophysical aspects, missing out on cell biology and physiology) or in mutants of Arabidopsis (perfect for developmental aspects, missing out on cell biology and physiology). Inhibitor studies revealed that this localisation was depending on actin filaments, but not on microtubules. The overexpression of the *Nt*TPC1A fusion protein was accompanied by subtle, but specific phenotypic responses, such as impaired cell elongation (depending on auxin), reduced intracellular calcium content (rescued by supplementation of calcium to the medium), partial resistance to the calcium-channel blocker gadolinium, and a higher sensitivity to the cell-death inducing protein harpin from the phytopathogenic bacterium *Erwinia amylovora*. To what extent these phenotypic changes can be attributed to calcium alone, can be discussed in the light of a high permeability to other ions, such as potassium (for a discussion see Blatt 2024). Keeping this caveat in mind, we will discuss the following questions on the level of calcium patterns: How does a channel residing in the tonoplast, modulate events that are localised at the plasma membrane? What can we learn on the role of calcium homeostasis for auxin-dependent growth? What is the link between actin filaments and *Nt*TPC1A? How can we interpret the role of *Nt*TPC1A in pathogen-induced cell death?

### Localisation at the tonoplast versus function at the plasma membrane

The subcellular localisation of TPC1 channels has been controversial. Patch-clamp data (Peiter et al. 2005; Dadacz-Narloch et al. 2013) initially suggested a localisation at the tonoplast, while transient expression of a GFP fusion in onion cells showed a signal that was clearly at the plasma membrane (Kurusu et al*.*, 2004; Wang et al. 2005). Expression of the Arabidopsis TPC1 homologue in tobacco BY-2 was claimed to show a plasma membrane localisation (Kawano et al. 2004), but the quality of the images was too poor to support this claim. In the same study, release of cytosolic Ca^2+^ was shown to be blocked by aluminium ions. While we can confirm inhibition of cell growth by aluminium (Fig. 7A), we did not observe any significant difference between WT and overexpressor, contrasting with the situation observed for Gd^3+^ (Fig. 7B). Thus, the alleged localisation at the plasma membrane might be an artifact, though. First, localisation in heterologous hosts may differ due to different interaction partners. Second, transient expression relies on high-copy vectors such that interaction partners needed for correct localisation might become oversaturated. Third, biolistics relies on breached membrane integrity and the subsequent healing process is expected to interfere with the natural dynamics and architecture of the endomembrane system. Our current approach uses a homologous system—tobacco BY-2 cells hosting the *Nt*TPC1A from tobacco BY-2 cells—and stable expression, selecting for those cells, where physiology has been preserved to an extent that these cells can cycle and expand at a normal pace. Using spinning-disc confocal microscopy, we find that the GFP signal reporting *Nt*TPC1A is on the tonoplast (Fig. 3). Even a residual plasma membrane localisation would become visible upon protoplasting. There is none to be seen (Fig. 4). To reach a quantitative estimate, we conducted a double labelling experiment where the plasma membrane was labelled by a short pulse of FM4-64 (Suppl. Fig. S1). This quantification, too, shows that only a minute fraction of the *Nt*TPC1A-GFP signal is found at the plasma membrane (around 5% of the signal seen in the tonoplast). We conclude that *Nt*TPC1A is localised to the tonoplast, while localisation at the plasma membrane is definitely negligible. It should be mentioned that also recent electrophysiological measurements discount an activity of this channel with its specific kinetic properties at the plasma membrane (for review see Blatt 2024).

However, this does not necessarily mean that the functions conveyed by *Nt*TPC1A are confined to the tonoplast. In fact, we find several indications for interactions with events that take place at the plasma membrane. One of the most astonishing findings is the significant reduction of cellular content of Ca^2+^ in the *Nt*TPC1A overexpressor, and the rescue of this overexpression phenotype by complementing extracellular Ca^2+^ (Fig. 2B). Likewise, the response to transportable auxin and the auxin-efflux inhibitor NPA is altered (Fig. 5, Suppl. Fig. S2), as well as the responses to the three-valent ion gadolinium that is unlikely to penetrate through the plasma membrane (Fig. 7B), or the response, mainly of cycling cells, to the bacterial elicitor harpin (Fig. 8B), that acts on the membrane located NADPH oxidase Respiratory burst oxidase Homologue (Eggenberger et al. 2017).

Cross-talk of vacuolar calcium transporters with events at the plasma membrane is not confined to the current case. For instance, the basal immunity triggered by the bacterial elicitor flagellin, is triggered by a calcium influx at the plasma membrane mediated by a heterodimer of the Cyclic-Nucleotide Gated Channel (CNGC) family (Tian et al. 2019). Although this event is undisputedly localised to the plasma membrane, it is strongly dependent on the activity of specific members of the autoinhibited calcium ATPases (ACA) family that are undisputedly located in the tonoplast (Hilleary et al. 2020). When these channels are knocked out, this leads to a stronger calcium influx in response to elicitation by flagellin. These specific CNGC members are endowed with a binding site for calmodulin. This binding site has been functionally validated and co-expression of calmodulin in oocytes was observed to inhibit CNGC activity. This means that their activity is gated by calmodulin (Tian et al. 2019).

Based on biophysical considerations derived from patch-clamp studies on Slow Vacuolar calcium channels, likely to be identical to TPC1 (Pottosin and Schoenknecht, 2007), this tonoplast channel has been proposed to act as major driver for release of Ca^2+^ from the vacuole into the cytoplasm, rather than as uptake channel. Overexpression of *Nt*TPC1A would be expected to activate calmodulin, which would then suppress CNGC-dependent calcium influx. This hypothesis would imply that the uptake of Ca^2+^ into the cell should be reduced in the overexpressor due to lower influx activity, and that this should be rescued by increasing Ca^2+^ levels in the external medium. This implication is matching well with our observation (Fig. 2B). However, the highly artificial conditions that had to be established to measure export activity from the vacuole have been critically discussed in Hedrich et al. (2018). On the other hand, binding of Ca^2+^ to a cytosolic EF-hand in *Nt*TPC1A can activate Ca^2+^ export from the vacuole, establishing a non-linear amplification loop. Thus, while several scenarios can be conceived, how overexpression of a vacuolar transporter might alter ion flux at the plasma membrane, the mechanistic details have remained an open question, as stated in a recent review (Hedrich et al. 2023): ‘How is the coupling of plasma membrane and vacuolar membrane electrical signaling achieved?’ Such global effects, extending beyond the site of localisation of calcium transport, are not uncommon, though. For instance, mutations of endomembrane calcium ATPases were shown to modulate ion fluxes at the plasma membrane, probably to incomplete re-sequestration of cytosolic calcium (Jezek et al. 2021).

### *Nt*TPC1A modulates the actin-dependent calcium-auxin loop

Our study draws two links from the *Nt*TPC1A overexpression towards auxin signalling and transport: These cells are shorter and wider and their response to superoptimal concentrations of the transportable auxin IAA is more pronounced (Fig. 5). The steady-state concentration of endogenous auxin is certainly much lower, due to strong catabolism by an active dioxygenase (Müller et al. 2021). We did not determine, to what extent the residual IAA is elevated over the roughly 5 nM measured in those experiments. However, we have observed in the past that, at least in dark-grown cells, exogenous IAA can modify developmental responses over several day (Maisch and Nick 2007; Huang et al. 2017). On the other hand, overexpression of *Nt*TPC1A qualitatively changes the response of cell width to NPA, an inhibitor of polar auxin transport—these cells become slightly, but significantly slimmer, while the cells of the non-transformed wild type retain their width (Suppl. Fig. S2). These differences in terms of auxin response and transport are accompanied with a clear localisation of the *Nt*TPC1A signal with actin filaments (Fig. 6), and a loss of this localisation, when actin is eliminated, while elimination of microtubules has no effect on *Nt*TPC1A (Fig. 7).

A potential link between Ca^2+^ and auxin has been a constant theme in auxin research, since a classic experiment showed that polar auxin transport is blocked by chelators, but can be rescued by supplemented exogenous Ca^2+^ (Dela Fuente and Leopold 1973), demonstrating that Ca^2+^ is necessary for the transport of auxin. Interestingly, this interrelation is subject to pronounced oscillation—not only does auxin induce oscillations of cytosolic Ca^2+^ and pH that run with a period of 20–30 min (Felle 1988), but also auxin transport itself is oscillating with a similar period (Hertel and Flory 1968). The missing link to explain these oscillations seems to be actin: Actin filaments can remodel between a highly dynamic cortical network and stable transvacuolar cables depending on the presence of auxin and this feeds back on the activity of auxin transport. The dynamic cortical network sustains efficient efflux of auxin, while the stable cables restrain this transport (Nick et al. 2009) leading to a self-referring feedback loop between auxin transport and actin remodelling with a period of 20–30 min as observed for both auxin transport and cytosolic Ca^2+^ (reviewed in Nick 2010). The mechanistic link could be actin-binding proteins, whose activity is regulated by Ca^2+^, such as profilin, LIM proteins, or villins (for review see Qian and Xiang 2019). On the other hand, actin nucleating proteins, such as Arp2/3 have been shown to control cytosolic Ca^2+^ (Zhao et al. 2013). If two factors are mutually intertwined both upstream and downstream, they tend to oscillate.

These oscillations could link to auxin signalling through the role actin filaments play for vacuolar expansion: Fissions or fusions of vacuoles are important for the inhibition of cell expansion by superoptimal concentrations of auxin and have been shown to depend on actin remodelling (Scheuring et al. 2016). The effect of *Nt*TPC1A overexpression on auxin-dependent growth inhibition might be caused in a similar way by modulating the self-referring loop between auxin, Ca^2+^, and actin.

### A role for *Nt*TPC1A in defence-related cell death?

When *Nt*TPC1A-driven fluctuations of Ca^2+^ connects actin remodelling and auxin-dependent growth, it might as well interfere with other actin-related phenomena. Defence-related cell death is a prime candidate, because it is depending on actin remodelling (Chang et al. 2015), and at the same time on Ca^2+^ because the metacaspases that execute cell death, are activated by Ca^2+^ dependent auto-cleavage (van Midden et al. 2021). In fact, we observe that overexpression of *Nt*TPC1A promotes the mortality response to harpin, an elicitor from the phytopathogenic bacterium *Erwinia amylovora*, the causative agent of fire blight in apple trees. This bacterium is necrotrophic and uses harpin to evoke an illegitimate hypersensitive response, which would be useful against biotrophic pathogens, but is completely inappropriate in this context (Wei et al. 1992). To induce cell death, harpin requires actin, because elimination of actin by Latrunculin B can intercept the effect to this elicitor (Chang et al. 2015). The remodelling activity of several actin-binding proteins, such as profilin or villin, but also the nucleating Actin-Related Protein 2/3, is regulated by Ca^2+^ (for review see Li et al. 2015). Harpin not only triggers the plasma membrane located NADPH oxidase, which will generate an oxidative burst in the apoplast, evoking Ca^2+^ influx (Chang and Nick 2012), which is expected to deploy these calcium-dependent actin regulating proteins. The overexpression of *Nt*TPC1A should amplify the levels of cytosolic Ca^2+^ and, therefore, amplify the response to harpin. This does not imply, however, that harpin induces a hypersensitive response by activation of harpin. To demonstrate that, one would need to be able to knock down *NtTCP1A* in the WT and show that the responsiveness to harpin is lost (an experiment that is far from trivial to conduct in BY-2, because neither induced antisense, nor genome editing are working well in this system). Thus, we can only state that *Nt*TPC1A modulates the response to harpin.

The link of a tonoplast-located calcium transporter with actin shifts the focus on the role of actin for vacuole integrity. Defence-related cell death recruits components that are also used for autophagy (Hofius et al. 2009). A component of the actin-nucleation complex, NAP1, is required for autophagosome formation (reviewed in Wang et al. 2020). This seems to be specific for defence, since other forms of autophagy, such as salinity induced necrosis, can proceed even after the entire actin cytoskeleton has been eliminated either pharmacologically or by genetic interference (Zheng et al. 2019).

Ca^2+^ is directly regulating the structure of the actin nucleation complex (Nolen et al. 2004). On the other hand, this complex mediates stress-induced release of Ca^2+^ from internal stores (Zhao et al. 2013), establishing a self-amplifying feedback loop. A straightforward hypothesis would explain the promoted hypersensitive reaction of the *Nt*TPC1A overexpressor via the stimulation of this feedback loop that would also promote the recruitment of autophagy-related components and, thus, boost the execution of defence-related cell death.

### Outstanding questions

Ca^2+^ is ubiquitous, yet signalling needs to be specific. The molecular nature of Ca^2+^cannot be the reason for this specificity leading to the concept of Ca^2+^signatures (Knight et al. 1991). The impact of temporal signatures has been well established and clearly implies integration over time. However, there are also spatial signatures—whether Ca^2+^ is present at the plasma membrane, at the interface between mitochondria and endoplasmic reticulum, or at the tonoplast, makes a difference. We still understand very little about such spatial signatures. However, the current study provides evidence that the abundance of a channel at the tonoplast can modulate signalling events at the tonoplast, a phenomenon that has been reported for other calcium channels as well (Tian et al. 2019). Also, this type of signature implies integration—this time integration over space. Is this integration conveyed by a gradient of the signal, Ca^2+^, which would require a global de-regulation of this signal, which is otherwise tightly regulated. Or is the regulation upstream of Ca^2+^, which would allow very localised release of Ca^2+^ in one site of the cell, depending on the calcium state of a localised site somewhere else? The phenotypes reported in the current work are likely to be caused by the changes in Ca^2+^ levels or distribution. However, at this stage it cannot concluded that these effects are caused by other ions that might be perturbed by the overexpression. Therefore, approaches to visualise and to manipulate Ca^2+^ levels locally will be required. Fluorescent reporters, such as cameleon or GCaMP5 might be tried to monitor subcellular Ca^2+^ distribution, with the caveat that these probes, by sequestration of Ca^2+^ can interfere with calcium homeostasis. Fluorescent Ca^2+^ probes, such as Fluo-4FF, might provide alternatives. Likewise, aequorin cells might be useful, because, here, the timing can be defined by adding the substrate coelenterazine. This temporal control was crucial for the discovery of temporal calcium signatures oin aequorin plants (Knight et al. 1991), in aequorin-expressing cells it might help to visualise its spatial signatures.

## Supplementary Information

Below is the link to the electronic supplementary material.

ESM1 Suppl. Fig. S1: Dual visualisation of *Nt*TPC1A-GFP at the tonoplast and the plasma membrane, labelled by a brief pulse (~ 1 min) with 5 µM of FM4-64 and quantification of cross-localisation of *Nt*TPC1A-GFP at the plasma membrane. Intensity profiles for the green and the red channel were collected along a probing line. A representative example of the line, the two channels and the collected profiles are shown. Peak heights in the two channels were used to estimate channel bleed factors and cross-localisation ratios. Data represent mean and standard errors for cross-localisation from twelve individual cells (PPTX 370 KB)

ESM2 Suppl. Fig. S2: Phenotype of cells overexpressing *Nt*TPC1A-GFP (*Nt*TPC1A-GFPox, grey bars) as compared to non-transformed tobacco BY-2 cells (WT, white bars) as assessed at the end of one cultivation cycle (7 d) without or with 10 µM of the auxin transport inhibitor 1-naphthyl-phthalamic acid (NPA) supplemented indole acetic acid (IAA). **A** Cell length, **B** cell width, **C** aspect ratio. **D** schematic visualisation of the shape change in response to IAA. Data points represent mean and standard error from at least 1500 individual cells obtained from three independent experimental series. Statistical significance of differences was tested by a Student t-test with ns non-significant, and ** significant at *P* < 0.01 (PPTX 54.4 KB)

ESM3 Supplemental Figure S3: Dose–response relation for cell growth (**A**) and mortality (**B**) over the concentration of NaCl in non-transformed tobacco BY-2 cells (WT) and cells overexpressing *Nt*TPC1A-GFP (*Nt*TPC1A-GFPox). Growth is given as Packed Cell Volume relative to the non-transformed wild type grown under control conditions measured at the end of the culture cycle, at day 7. Data for PCV represent mean and standard error from three independent experimental series, mortality was scored from a population of 500 individual cells. Statistical significance of differences was tested by a Student t-test with ns non-significant, * significant at *P* < 0.05, and ** significant at *P* < 0.01 (PPTX 44.9 KB)

ESM4 Supplemental Movie S4: Vacuolar remodelling in response to 9 mg^.^mL^−1^ of the bacterial elicitor harpin in representative cells treated at day 3 after subcultivation, in the cycling phase. The sequence covers 20 min in steps of 3 min. Confocal sections were collected in the nuclear plane (AVI 1.17 MB)

ESM5 Supplemental Figure S5: Vacuolar remodelling in response to 9 mg^.^mL^−1^ of the bacterial elicitor harpin in comparison to a representative cell that was not treated at day 3 after subcultivation, in the cycling phase. Images show frames from Supplemental Movie S5 (PPTX 625 KB)

ESM6 Supplemental Table S1. Conditions for amplifying of NtNTTPC1 by RT-PCR (DOCX 16.2 KB)

## Data Availability

Data can be obtained from the author upon reasonable request.
