# Supplementary material for: Tobacco two-pore calcium channel 1a is localised at the tonoplast, but acts on events at the plasma membrane
Source: Protoplasma. 2025 Oct 2;263(2):423–38. doi: 10.1007/s00709-025-02118-1 (PMC12945979; doi:10.1007/s00709-025-02118-1)
Supplement: Supplementary file 1 — Suppl. Fig. S1: Dual visualisation of NtTPC1A-GFP at the tonoplast and the plasma membrane, labelled by a brief pulse (~ 1 min) with 5 µM of FM4-64 and quantification of cross-localisation of NtTPC1A-GFP at the plasma membrane. Intensity profiles for the green and the red channel were collected along a probing line. A representative example of the line, the two channels and the collected profiles are shown. Peak heights in the two channels were used to estimate channel bleed factors and cross-localisation ratios. Data represent mean and standard errors for cross-localisation from twelve individual cells (PPTX 370 KB) [file 709_2025_2118_MOESM1_ESM.pptx]

## Slide 1
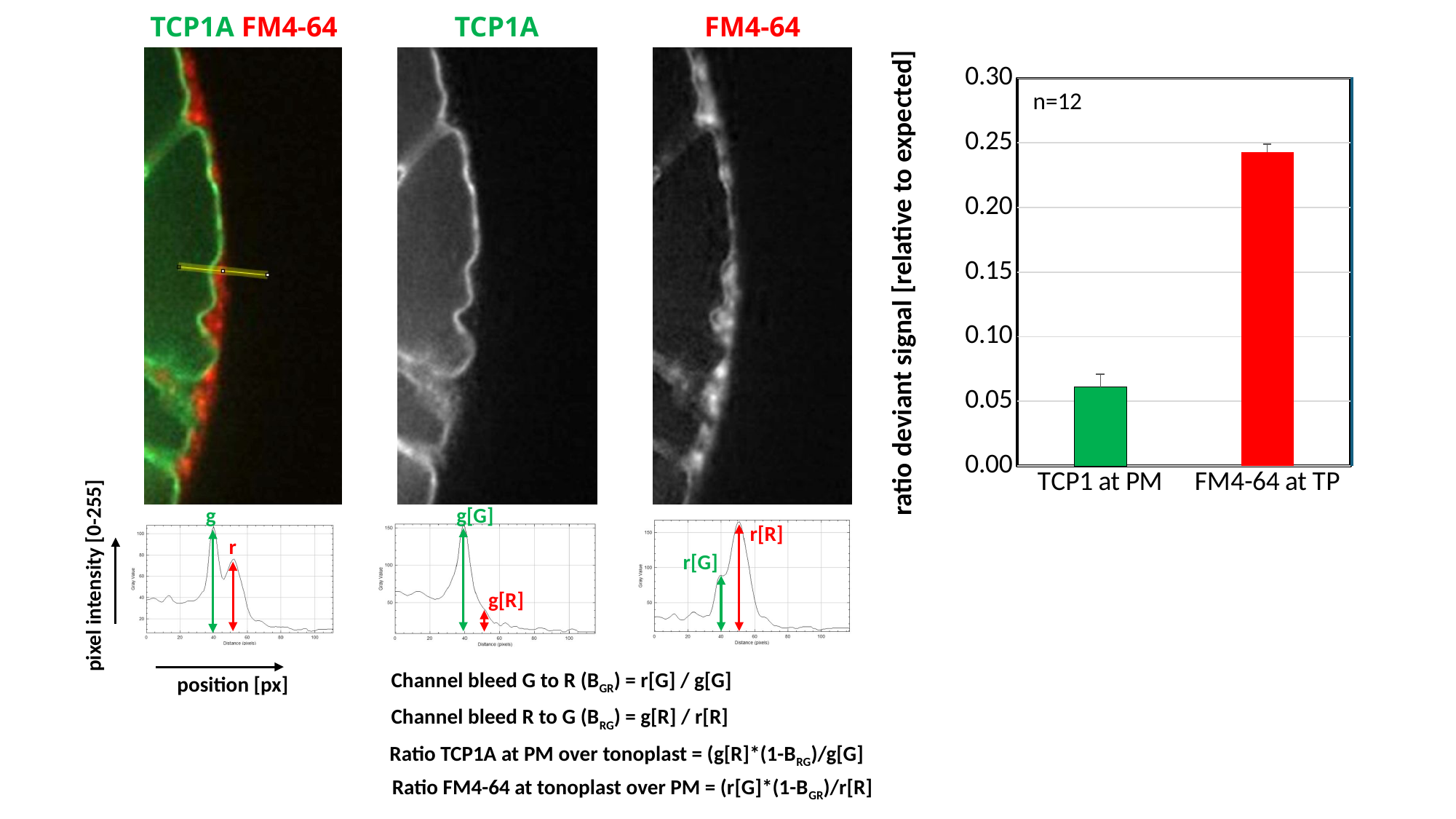

TCP1A FM4-64
TCP1A
FM4-64
### Chart
| Category | |
|---|---|
| TCP1 at PM | 0.061 |
| FM4-64 at TP | 0.243 |n=12
ratio deviant signal [relative to expected]
g[G]
g
r[R]
r
r[G]
pixel intensity [0-255]
g[R]
Channel bleed G to R (BGR) = r[G] / g[G]
position [px]
Channel bleed R to G (BRG) = g[R] / r[R]
Ratio TCP1A at PM over tonoplast = (g[R]*(1-BRG)/g[G]
Ratio FM4-64 at tonoplast over PM = (r[G]*(1-BGR)/r[R]
