# Supplementary material for: Tobacco two-pore calcium channel 1a is localised at the tonoplast, but acts on events at the plasma membrane
Source: Protoplasma. 2025 Oct 2;263(2):423–38. doi: 10.1007/s00709-025-02118-1 (PMC12945979; doi:10.1007/s00709-025-02118-1)
Supplement: Supplementary file 2 — Suppl. Fig. S2: Phenotype of cells overexpressing NtTPC1A-GFP (NtTPC1A-GFPox, grey bars) as compared to non-transformed tobacco BY-2 cells (WT, white bars) as assessed at the end of one cultivation cycle (7 d) without or with 10 µM of the auxin transport inhibitor 1-naphthyl-phthalamic acid (NPA) supplemented indole acetic acid (IAA). A Cell length, B cell width, C aspect ratio. D schematic visualisation of the shape change in response to IAA. Data points represent mean and standard error from at least 1500 individual cells obtained from three independent experimental series. Statistical significance of differences was tested by a Student t-test with ns non-significant, and ** significant at P < 0.01 (PPTX 54.4 KB) [file 709_2025_2118_MOESM2_ESM.pptx]

## Slide 1
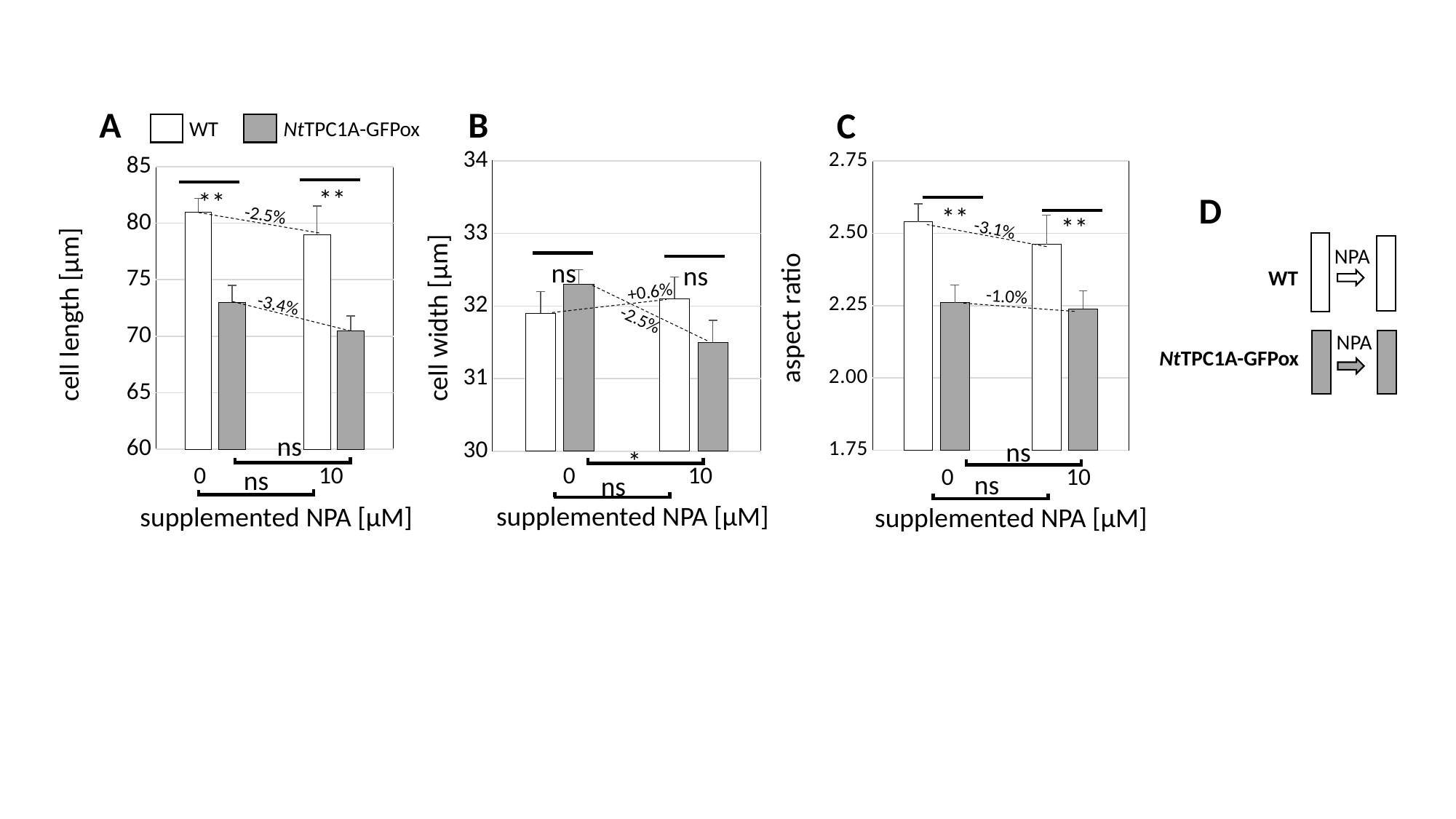

A
B
C
WT
NtTPC1A-GFPox
### Chart
| Category | WT | TPC1aox |
|---|---|---|
| control | 31.9 | 32.3 |
| NPA | 32.1 | 31.5 |
### Chart
| Category | WT | TPC1aox |
|---|---|---|
| | 2.5391849529780566 | 2.2600619195046443 |
| | 2.4610591900311527 | 2.238095238095238 |
### Chart
| Category | WT | TPC1aox |
|---|---|---|
| control | 81.0 | 73.0 |
| NPA | 79.0 | 70.5 |**
**
D
**
-2.5%
**
-3.1%
NPA
ns
ns
WT
+0.6%
-1.0%
-3.4%
cell length [µm]
aspect ratio
cell width [µm]
-2.5%
NPA
NtTPC1A-GFPox
ns
ns
*
0
10
0
10
0
10
ns
ns
ns
supplemented NPA [µM]
supplemented NPA [µM]
supplemented NPA [µM]
