# Supplementary material for: Tobacco two-pore calcium channel 1a is localised at the tonoplast, but acts on events at the plasma membrane
Source: Protoplasma. 2025 Oct 2;263(2):423–38. doi: 10.1007/s00709-025-02118-1 (PMC12945979; doi:10.1007/s00709-025-02118-1)
Supplement: Supplementary file 3 — Supplemental Figure S3: Dose–response relation for cell growth (A) and mortality (B) over the concentration of NaCl in non-transformed tobacco BY-2 cells (WT) and cells overexpressing NtTPC1A-GFP (NtTPC1A-GFPox). Growth is given as Packed Cell Volume relative to the non-transformed wild type grown under control conditions measured at the end of the culture cycle, at day 7. Data for PCV represent mean and standard error from three independent experimental series, mortality was scored from a population of 500 individual cells. Statistical significance of differences was tested by a Student t-test with ns non-significant, * significant at P < 0.05, and ** significant at P < 0.01 (PPTX 44.9 KB) [file 709_2025_2118_MOESM3_ESM.pptx]

## Slide 1
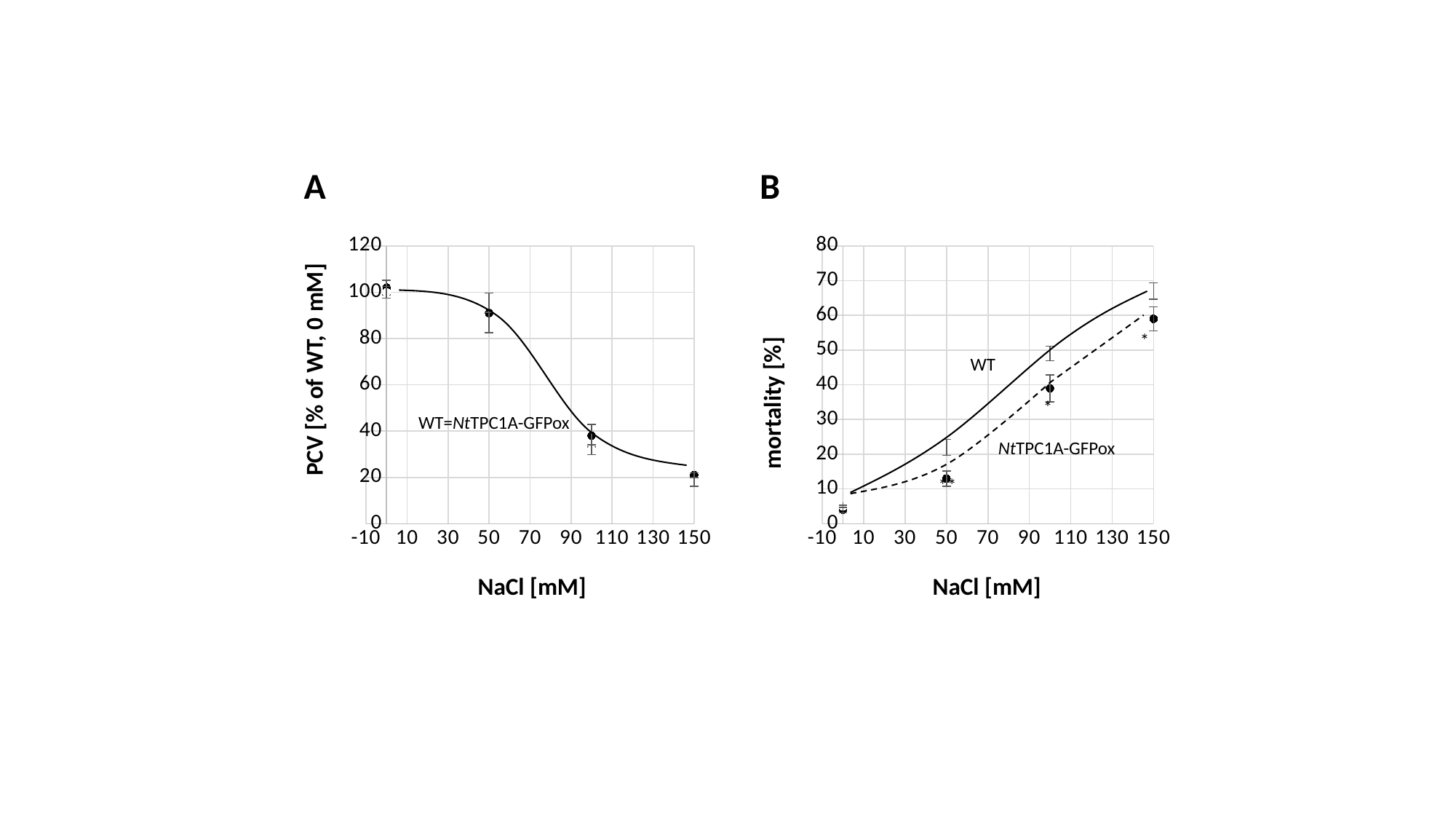

A
B
### Chart
| Category | WT | TPC1a ox |
|---|---|---|
### Chart
| Category | WT | TPC1a ox |
|---|---|---|
*
WT
PCV [% of WT, 0 mM]
mortality [%]
*
WT=NtTPC1A-GFPox
NtTPC1A-GFPox
**
NaCl [mM]
NaCl [mM]
