# Supplementary figures and images for: Tobacco two-pore calcium channel 1a is localised at the tonoplast, but acts on events at the plasma membrane
Source: Protoplasma. 2025 Oct 2;263(2):423–38. doi: 10.1007/s00709-025-02118-1 (PMC12945979; doi:10.1007/s00709-025-02118-1)

## Slide 1
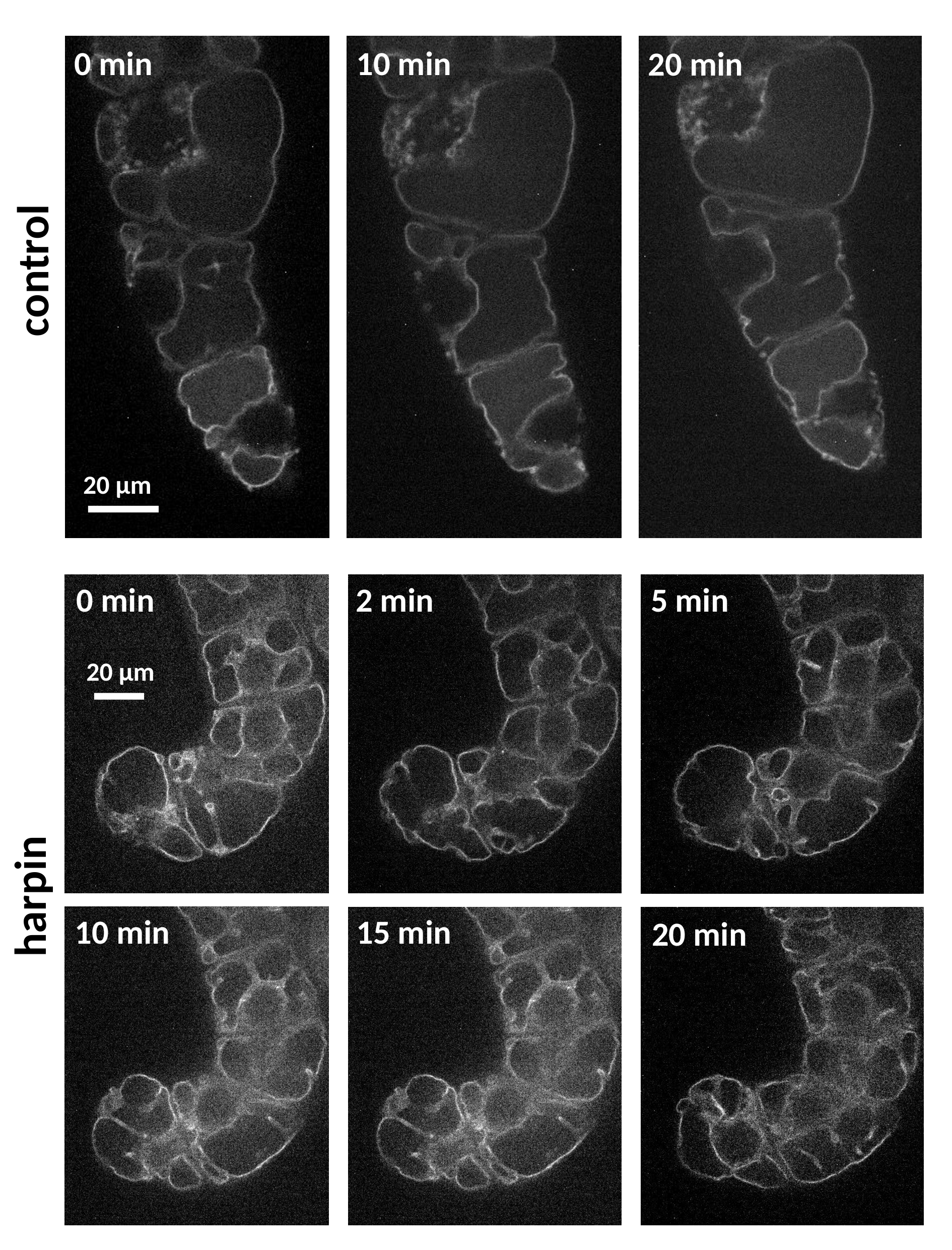

0 min
10 min
20 min
control
20 µm
0 min
2 min
5 min
20 µm
harpin
10 min
15 min
20 min

Supplement: Supplementary file 5 — Supplemental Figure S5: Vacuolar remodelling in response to 9 mg.mL−1 of the bacterial elicitor harpin in comparison to a representative cell that was not treated at day 3 after subcultivation, in the cycling phase. Images show frames from Supplemental Movie S5 (PPTX 625 KB) [file 709_2025_2118_MOESM5_ESM.pptx]
