# Supplementary material for: Tobacco two-pore calcium channel 1a is localised at the tonoplast, but acts on events at the plasma membrane
Source: Protoplasma. 2025 Oct 2;263(2):423–38. doi: 10.1007/s00709-025-02118-1 (PMC12945979; doi:10.1007/s00709-025-02118-1)
Supplement: Supplementary file 6 — Supplemental Table S1. Conditions for amplifying of NtNTTPC1 by RT-PCR (DOCX 16.2 KB) [file 709_2025_2118_MOESM6_ESM.docx]

**Supplemental Table S1.** Conditions for amplifying of NtNTTPC1 by RT-PCR.

| **Oligonucleotide primers** | | |
| --- | --- | --- |
| *Nt*TPC1Afw | 5’-GGGGACAAGTTTGTACAAAAAAGCAGGCTTCATGGAAGAATATCTACTGTCAGGG-3’ | |
| *Nt*TPC1A-rev | 5’-GGGGACCACTTTGTACAAGAAAGCTGGGTCTGGATTCTCATTGGAGCATTCTG-3’ | |
| q*Nt*TPC1A-fw | 5’-GAGTAGTAATTCGGGTCGGACTCG-3’ | |
| q*Nt*TPC1A-rev | 5’-GCAGACCAATGCCATCTTCAGC-3’ | |
| GAPDH-fw | 5’-ACAAATTGCCTTGCTCCCTTGGC-3’ | |
| GAPDH-rev | 5’- CCTCCAGTCCTTGGCTGATGG-3’ | |
| L25-fw | 5’- GTTGCCAAGGCTGTCAAGTCAGG-3’ | |
| L25-rev | 5’- 5’- GTTGCCAAGGCTGTCAAGTCAGG-3’ | |
| **PCR mix** | | |
| template cDNA | | 1 µL |
| forward primer (10 µM) | | 2 µL |
| reverse primer (10 µM) | | 2 µL |
| dNTPs (10 mM) | | 1 µL |
| High Fidelity buffer (5x) | | 10 µL |
| Phusion Polymerase (New England Biolabs) | | 0.5 µL |
| betaine (5 M) | | 5 µL |
| DMSO (100%) | | 2 µL |
| double distilled water | | 26.5 µL |
| total volume | | 50 µL |
| **PCR conditions** | | |
| pre-heating | | 98°C, 30 s |
| amplification  38 cycles | denaturation | 98°C, 10 s |
|  | annealing | 56°C, 35 s |
|  | extension | 72°C, 45 s |
| final extension | | 72°C, 7 min, then hold on 4°C |
